# Epidemiology of endometriosis in Kazakhstan: a national population-based cohort analysis (2014–2019) using data from the national electronic healthcare system

**DOI:** 10.3389/fmed.2024.1436458

**Published:** 2025-01-07

**Authors:** Gulzhanat Aimagambetova, Yesbolat Sakko, Talshyn Ukybassova, Milan Terzic, Aizada Marat, Nazira Kamzayeva, Arnur Gusmanov, Gulnur Zhakhina, Sauran Yerdessov, Kamilla Mussina, Dmitriy Syssoyev, Abduzhappar Gaipov

**Affiliations:** ^1^Department of Surgery, School of Medicine, Nazarbayev University, Astana, Kazakhstan; ^2^Department of Medicine, School of Medicine, Nazarbayev University, Astana, Kazakhstan; ^3^Clinical Academic Department of Women’s Health, CF “University Medical Center”, Astana, Kazakhstan; ^4^Department of Obstetrics and Gynecology #1, NJSC “Astana Medical University”, Astana, Kazakhstan; ^5^Clinical Academic Department of Internal Medicine, CF “University Medical Center”, Astana, Kazakhstan

**Keywords:** endometriosis, infertility, epidemiology, prevalence, Kazakhstan

## Abstract

**Background:**

Endometriosis is a condition affecting reproductive-age women and associated with dysmenorrhea, pelvic organs dysfunction, pelvic pain, and infertility. The real epidemiology of endometriosis remains underestimated. No data are available on prevalence of endometriosis in Kazakhstan. Therefore, the aim of this was to investigate the epidemiology, complications, surgical management approach, and outcomes of endometriosis in Kazakhstan by analyzing large-scale Kazakhstani healthcare data from the Unified Nationwide Electronic Health System (UNEHS).

**Methods:**

A population-based study among women with endometriosis treated in any healthcare setting of the Republic of Kazakhstan during the period of 2014–2019 was performed. The International Classification of Diseases (ICD) 10th edition was used to retrieve data on endometriosis (“N80” and “N97”). ICD 9th edition’s procedural codes were utilized to retrieve information on surgical procedures performed to manage patients with endometriosis.

**Results:**

In total, 7,682 records of women diagnosed with endometriosis were analyzed from all Kazakhstani regions. The overall prevalence of endometriosis among Kazakhstani female population was 0.12%, with 50.1% of them suffering from endometriosis of the uterus, 34.5% with ovarian endometriosis, and 9.5% with endometriosis of pelvic peritoneum. The most affected group was reproductive-age women (25–44 years old). Endometriosis rates were higher among women of 35–39, 40–44, and 45–49 years old age groups – 0.4 per 1000 women of corresponding age. The most common procedures performed for surgical management were laparoscopic cystectomy and closed biopsy of the uterus, 16.4 and 13.5%, respectively.

**Conclusion:**

Among all registered cases of endometriosis, ovarian endometriosis is the most prevalent condition. However, the analysis of the UNEHS records on endometriosis reveals incomplete and inconsistent registration of the disease, which results in the underestimation of the disease’s real burden. Clinical specialist and health authorities in Kazakhstan must work to ensure the endometriosis proper diagnosis end registration to improve the disease management and outcomes.

## 1 Background

Endometriosis is a chronic benign gynecological disease affecting reproductive-age women and associated with dysmenorrhea, pelvic organs’ dysfunction, pelvic pain, and infertility (1–6). Although visual identification is often used for clinical verification of endometriosis, the definitive diagnosis requires histological confirmation of the ectopic endometrial glands and stroma presence outside of the uterine cavity (3, 6). Adenomyosis is characterized by the invasion of endometrial glands and stroma within the myometrium (7).

There are different classifications systematizing endometriosis nomenclature: based on localization, extension, and depth of the ectopic endometrial glands (1–3). The typical localization of endometriosis is pelvic organs. The most common types of pelvic endometriosis are ovarian endometriotic cysts and superficial peritoneal lesions (1, 3, 4). Deep infiltrating lesions are less common and defined as lesions with more than 5 mm depth of invasion into the organs’ stromal tissues or beneath the peritoneum (1, 3).

According to the available statistical data, endometriosis affects 5–10% of reproductive-age women worldwide (3). However, due to the heterogeneity of endometriosis and multiple definitions used to describe the disease, due to the difference in the disease reporting and registration, the prevalence of endometriosis remains underestimated (6, 8). Moreover, according to different reports, the prevalence of endometriosis is even higher among infertile women and varies from 25 to 60% (1, 6, 9–11). Among women suffering from chronic pelvic pain, the prevalence of ovarian cysts and deep endometriosis were reported in over 25 and 1–5% of cases, respectively (6). Moreover, according to a recent study subtle endometriosis was reported in 40% of asymptomatic women (6). These data make it evident that the estimation of the epidemiology of endometriosis is important for female health care.

Endometriosis and adenomyosis interfere with fertility and pregnancy course via different mechanisms: disruption of pelvic anatomy, and affect oocyte release, uptake, or transport through the fallopian tubes (12, 13). Furthermore, chronic pelvic inflammation with prostaglandins and various inflammatory cytokines production could potentially impact the physiology of ovulation, conception, embryo migration, and implantation (13). The recent meta-analysis reported increased sporadic and recurrent pregnancy loss rates and reduced pregnancy and live birth rates in women with endometriosis and adenomyosis (13, 14). Thus, approximately 10–25% of women with endometriosis-associated infertility require treatment with assisted reproductive technology (ART) (1, 11).

The Republic of Kazakhstan is a Central Asian country with a population of around 20 million (15–17). According to the Kazakhstani National Agency for Statistics, females account for 52% of the population with 51% belonging to the reproductive-age group (2, 17–20). To date no studies have been done on the epidemiology of endometriosis in Kazakhstan, thus, no data is available on the incidence, prevalence, and complications of the disease. At the same time, according to available resources, the prevalence of infertility is high in the Republic of Kazakhstan (19–22). Based on recent publications, the frequency of infertility varies between 12 and 15.5% (20–22). Assuming a contribution of endometriosis to female gynecological morbidity and the prevalence of endometriosis-associated infertility, it is important to estimate the disease epidemiology. Thus, considering the high prevalence of infertility and the absence of statistical data on endometriosis and its contribution to the pool of infertility, this study’s aim was to investigate the epidemiology, complications, surgical management approach, and outcomes of endometriosis in Kazakhstan by analyzing large-scale Kazakhstani healthcare data from the Unified National Electronic Healthcare System (UNEHS).

## 2 Materials and methods

### 2.1 Study design and population

The study population consisted of patients who were hospitalized with endometriosis in any Kazakhstani clinical setting during the period of 2014–2019. The data was retrieved from UNEHS inpatient registry that was introduced at the end of 2013 to unify healthcare data storage through the country healthcare system (23). The International Classification of Diseases (ICD), 9th^1^ and 10th (see text footnote 1) editions were used for coding surgeries and diagnoses (primary and complication), respectively.

### 2.2 Patient selection and definitions

**FIGURE 1:**
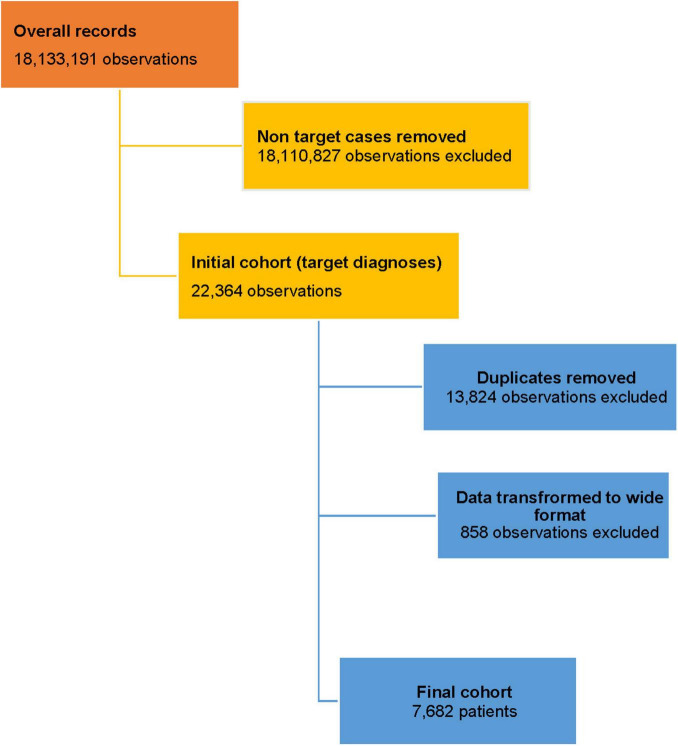
Data selection flow chart.

The initial dataset consists of overall 22,364 medical records of women registered with endometriosis and infertility (ICD-10 codes “N80” and “N97”). ICD-9 codes through “65” and “66” were reviewed to identify the surgical procedures performed to manage patients with endometriosis. Data cleaning was performed using unique patient ID, which links data throughout the UNEHS database. The final dataset included 7,682 patients. The detailed patient selection process is depicted in Figure 1.

### 2.3 Statistical analysis

The study involved descriptive and bivariate analyses to explore the association of demographic characteristics and diagnosis among the participants. Categorical variables were described by numbers and percentages, and their relationship with diagnosis was tested using Pearson’s chi-square test. Age was described by median and interquartile range (IQR), and the difference among groups was tested using Kruskal-Wallis test. Two-sided *p*-values less than 0.05 indicated statistical significance for all tests. Prevalence of endometriosis per 1000 women of specific age groups was calculated using population statistics according to the National Agency for Statistics and Strategic Planning of the Republic of Kazakhstan (18). Stata 16 MP2 software was used for data processing and statistical analysis (24). More details about the data and methodology were published previously (23).

### 2.4 Ethical approval

The study was conducted in compliance with the Declaration of Helsinki, and approved by the Nazarbayev University Institutional Review Ethics Committee (protocol reference NU-IREC 490/18112021). Exemption from informed consent has been granted due to the retrospective nature of the study and anonymized data analysis. No individual patients’ information was reported in this study.

## 3 Results

### 3.1 Study subjects description

The study database included overall 30,221,986 with 18,133,191 female patients’ records available. Out of total female patients, 22,364 (0.12%) had an endometriosis diagnosis recorded between 2014 and 2019. In total, for the 6-year period included in this investigation, 7,682 women were diagnosed with endometriosis of any localization and registered in the UNEHS (Figure 1). These records of patients with endometriosis were identified and analyzed in the national electronic database from all Kazakhstani regions (Figure 1).

The study subjects’ social and demographic characteristics are presented in Table 1. The age of women registered with endometriosis ranged from 17 to 54 years, and the median age of the participants was 37.0 (IQR 30.0–45.0) years. The majority of women diagnosed with endometriosis were of reproductive age, between 25 and 44 years (66.4%).

**TABLE 1:** Socio-demographic characteristics of the study subjects (2014–2019).

Variable	Overall, *N* (%)	Rate per 1000 women of corresponding age	Diagnosis (ICD-10), *N* (%)									*p*-value
			**N80.0**	**N80.1**	**N80.2**	**N80.3**	**N80.4**	**N80.5**	**N80.6**	**N80.8**	**N80.9**	
Age, median (IQR)	37.0 (30.0, 45.0)	–	43.0 (36.0, 48.0)	31.0 (27.0, 38.0)	34.0 (28.0, 41.0)	31.0 (27.0, 36.0)	32.0 (27.0, 38.0)	37.0 (29.0, 44.0)	35.0 (31.0, 37.0)	36.0 (30.0, 42.0)	34.0 (29.0, 40.0)	<0.001*
**Age groups**												<0.001**
15–19	65 (0.8%)	0.02	5 (0.1%)	44 (1.7%)	1 (1.2%)	12 (1.7%)	0 (0.0%)	0 (0.0%)	0 (0.0%)	3 (1.5%)	0 (0.0%)	
20–24	519 (6.8%)	0.2	68 (1.8%)	345 (13.0%)	9 (11.1%)	74 (10.2%)	8 (6.6%)	0 (0.0%)	1 (7.7%)	11 (5.4%)	3 (13.6%)	
25–29	1,262 (16.4%)	0.3	267 (6.9%)	702 (26.5%)	18 (22.2%)	198 (27.3%)	35 (28.7%)	5 (33.3%)	1 (7.7%)	34 (16.7%)	2 (9.1%)	
30–34	1,348 (17.5%)	0.3	455 (11.8%)	578 (21.8%)	18 (22.2%)	206 (28.4%)	33 (27.0%)	3 (20.0%)	5 (38.5%)	44 (21.7%)	6 (27.3%)	
35–39	1,299 (16.9%)	0.4	595 (15.5%)	466 (17.6%)	12 (14.8%)	144 (19.8%)	24 (19.7%)	2 (13.3%)	4 (30.8%)	47 (23.2%)	5 (22.7%)	
40–44	1,200 (15.6%)	0.4	800 (20.8%)	278 (10.5%)	16 (19.8%)	58 (8.0%)	16 (13.1%)	2 (13.3%)	1 (7.7%)	25 (12.3%)	4 (18.2%)	
45–49	1,141 (14.9%)	0.4	902 (23.4%)	175 (6.6%)	5 (6.2%)	26 (3.6%)	4 (3.3%)	3 (20.0%)	1 (7.7%)	23 (11.3%)	2 (9.1%)	
≥50	847 (11.0%)	0.3	759 (19.7%)	60 (2.3%)	2 (2.5%)	8 (1.1%)	2 (1.6%)	0 (0.0%)	0 (0.0%)	16 (7.9%)	0 (0.0%)	
**Ethnicity**												<0.001**
Kazakh	4,085 (53.2%)	0.3	1,705 (44.3%)	1,642 (62.0%)	40 (49.4%)	451 (62.1%)	81 (66.4%)	10 (66.7%)	10 (76.9%)	133 (65.5%)	13 (59.1%)	
Russian	1,092 (14.2%)	0.3	572 (14.9%)	372 (14.0%)	16 (19.8%)	95 (13.1%)	15 (12.3%)	1 (6.7%)	2 (15.4%)	19 (9.4%)	0 (0.0%)	
Other	2,468 (32.1%)	0.3	1,562 (40.6%)	614 (23.2%)	25 (30.9%)	178 (24.5%)	26 (21.3%)	4 (26.7%)	1 (7.7%)	49 (24.1%)	9 (40.9%)	
Missing	37 (0.5%)	–	12 (0.3%)	21 (0.8%)	0 (0.0%)	2 (0.3%)	0 (0.0%)	0 (0.0%)	0 (0.0%)	2 (1.0%)	0 (0.0%)	
**Residence**												<0.001**
Rural	1,699 (22.1%)	0.3	923 (24.0%)	533 (20.1%)	19 (23.5%)	145 (20.0%)	16 (13.1%)	2 (13.3%)	2 (15.4%)	58 (28.6%)	1 (4.5%)	
Urban	5,983 (77.9%)	0.3	2,928 (76.0%)	2,116 (79.9%)	62 (76.5%)	581 (80.0%)	106 (86.9%)	13 (86.7%)	11 (84.6%)	145 (71.4%)	21 (95.5%)	
**Total**	**7,682 (100%)**	**0.3**	**3,851 (50.1%)**	**2,649 (34.5%)**	**81 (1%)**	**726 (9.5%)**	**122 (1.6%)**	**15 (0.2%)**	**13 (0.2%)**	**203 (2.6%)**	**22 (0.3%)**	

*Kruskal-Wallis.

**Pearson’s chi-squared. ICD-10 codes: N80.0, Endometriosis of uterus; N80.1, Endometriosis of ovary; N80.2, Endometriosis of fallopian tube; N80.3, Endometriosis of pelvic peritoneum; N80.4, Endometriosis of rectovaginal septum and vagina; N80.5, Endometriosis of intestine; N80.6, Endometriosis in cutaneous scar; N80.8, Other endometriosis; N80.9, Endometriosis, unspecified.

The ethnic distribution of patients with endometriosis includes 53.2% of women of the Kazakh ethnic group, 14.2% of the Russian ethnic group, and 32.1% of other ethnicities living in Kazakhstan. For 0.5% of the study subjects ethnicity was not reported in the UNEHS.

The distribution of endometriosis cases reported in the UNEHS was very unequal in different regions (Supplementary Table 1). The largest number of endometriosis cases was reported from the North-Kazakhstan region (23.8%, 1,826 cases), Astana city (17.7%, 1,361 cases), Almaty city (14.2%, 1,092), and East-Kazakhstan region (13.0%, 1,000). However, only 0.2% (19) of cases were found in Shymkent, one of the large cities in the country. Low numbers were also reported from the Turkestan region (2.5%, 191), (Supplementary Table 1).

The number of patients registered with endometriosis from urban areas was much higher than that of the rural ones – 77.9 and 22.1%, respectively (Table 1).

### 3.2 Incidence and rates of endometriosis (2014–2019)

**FIGURE 2:**
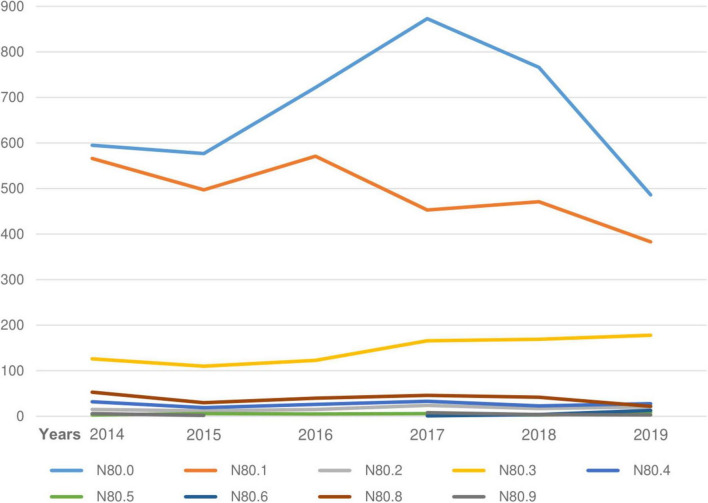
Incidence of endometriosis (2014–2019).

**FIGURE 3:**
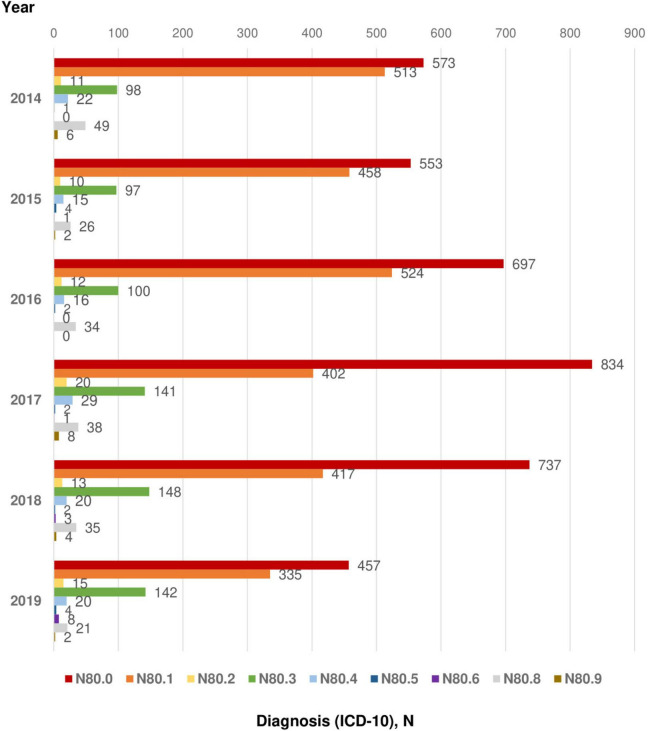
Main diagnosis, ICD-10. ICD-10 codes: N80.0 - Endometriosis of uterus; N80.1 - Endometriosis of ovary; N80.2 - Endometriosis of fallopian tube; N80.3 - Endometriosis of pelvic peritoneum; N80.4 - Endometriosis of rectovaginal septum and vagina; N80.5 - Endometriosis of intestine; N80.6 - Endometriosis in cutaneous scar; N80.8 - Other endometriosis; N80.9 - Endometriosis, unspecified.

Figure 2 and Table 1 report the incidence of endometriosis among women in Kazakhstan in 2014–2019 years. The incidence was equally distributed among women of the following age groups: 25–29 years old (16.4%), 30–34 years old (17.5%), 35–39 years old (16.9%), and 40–44 years old (15.6%). There was a gradual decrease in incidence after 45 years. A low number of cases reported in adolescent patients group (15–19 years old) – 0.8%, early reproductive age group (20–24 years old) −6.8%, and premenopausal age women (45–49 years old) - only 7.6% and- 14.9% of participants, respectively (Table 1). Figure 2 shows the incidence of endometriosis and its dynamics for the period of 6 years (2014–2019). Endometriosis of the uterus (ICD-10 code “N80.0”) was one of the most reported types of endometriosis – 50.1% (Figures 2, 3 and Table 1). Its incidence reporting increased in 2017 to 873 cases and dropped almost twice (486 cases) in 2019 (Figure 3). The second and third most prevalent types of endometriosis were endometriosis of ovaries (ICD-10 code “N80.1”) and endometriosis of pelvic peritoneum (ICD-10 code “N80.3”), 34.5 and 9.5%, respectively (Figure 2 and Table 1). However, the trends in the incidence of endometriosis of ovaries and peritoneal endometriosis were different: ovarian endometriosis cases are decreasing by 2019, while there was a slight increase in the incidence of peritoneal endometriosis through 2017–2019.

The endometriosis rates per 1000 women of corresponding age are shown in Table 1. While endometriosis rates were higher among women of 35–39, 40–44, and 45–49 years old age groups – 0.4 per 1000 women of corresponding age (Table 1). However, this indicator was low among women of early reproductive age groups (15–19 and 20–24 years old) - 0.02 and 0.2 per 1000 women of corresponding age, respectively.

### 3.3 Surgical procedures and invasive diagnostic manipulations performed for patients with endometriosis. Outcomes of disease management

**FIGURE 4:**
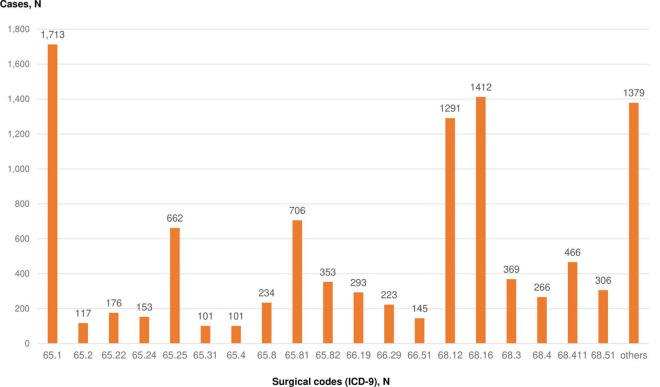
Diagnostic and surgical procedures performed for patients with endometriosis. ICD-9 codes: 65.1 – Laparoscopic cystectomy; 65.2 - Local excision or destruction of ovarian lesion or tissue; 65.22 – Ovarian wedge resection; 65.24 - Laparoscopic wedge resection of ovary; 65.25 - Other laparoscopic local excision or destruction of ovary; 65.31 - Laparoscopic unilateral oophorectomy; 65.4 -Unilateral salpingo-oophorectomy; 65.8 - Lysis of adhesions of ovary and fallopian tube; 65.81 - Laparoscopic lysis of adhesions of ovary and fallopian tube; 65.82 - Laparoscopic lysis of adhesions of ovary and fallopian tube; 66.19 - Other diagnostic procedures on fallopian tubes; 66.29 - Other bilateral endoscopic destruction or occlusion of fallopian tubes; 66.51 - removal of both tubes; 68.12 - Hysteroscopy; 68.16 - Closed biopsy of uterus (hysteroscopy with biopsy); 68.3—subtotal abdominal hysterectomy; 68.4 - total abdominal hysterectomy; 68.411 – Laparoscopic total hysterectomy; 68.51 - Laparoscopically assisted vaginal hysterectomy.

Diagnostic manipulations and surgical procedures performed for patients with endometriosis are shown in Figure 4. The most common surgical treatment procedure was laparoscopic cystectomy (ICD-9 code “65.1”) performed for 1,713 cases (16.4%). Other laparoscopic local excision or destruction of ovary (ICD-9 code “65.25”) and laparoscopic lysis of adhesions of ovary and fallopian tube (ICD-9 code “65.81”) were done for 662 (6.3%) and 706 (6.8%) patients, respectively (Figure 4). Some patients had two or more procedures performed simultaneously. The most common combined procedures are laparoscopic cystectomy with laparoscopic lysis of adhesions of ovary and fallopian tube and other laparoscopic local excision or destruction of ovary; hysteroscopy with closed biopsy of the uterus (hysteroscopy with biopsy).

As the most common diagnostic manipulation, closed biopsy of the uterus (ICD-9 code “68.16” - hysteroscopy with biopsy) in 1,412 cases (13.5%) and hysteroscopy (ICD-9 code “68.12”) - 1,291 cases (12.4%) were documented in the UNEHS database (Figure 4).

Out of all analyzed records, 69.2% of patients with endometriosis had a planned admission for surgical treatment, while 30.8% of patients were admitted via emergency route due to torsion or rupture of endometriotic cyst, bleeding due to endometriosis of the uterus (Table 2). The vast majority of patients with endometriosis were discharged after treatment (99.9%) with recovery or improvement (75.2 and 24.8%, respectively). There were no cases of mortality due to endometriosis registered in the UNEHS for the analyzed period (2014–2019).

**TABLE 2:** Outcomes of endometriosis treatment (2014–2019).

Variable	Overall, *N* (%)	Diagnosis (ICD-10), *N* (%)									*p*-value
		**N80.0**	**N80.1**	**N80.2**	**N80.3**	**N80.4**	**N80.5**	**N80.6**	**N80.8**	**N80.9**	
**Admission**											<0.001*
Emergency	2,368 (30.8%)	1,386 (36.0%)	762 (28.8%)	29 (35.8%)	134 (18.5%)	14 (11.5%)	3 (20.0%)	3 (23.1%)	34 (16.7%)	3 (13.6%)	
Planned	5,314 (69.2%)	2,465 (64.0%)	1,887 (71.2%)	52 (64.2%)	592 (81.5%)	108 (88.5%)	12 (80.0%)	10 (76.9%)	169 (83.3%)	19 (86.4%)	
**Outcome of stay**											
Discharge	7,672 (99.9%)	3,846 (99.9%)	2,644 (99.8%)	81 (100%)	726 (100%)	122 (100%)	15 (100%)	13 (100%)	203 (100%)	22 (100%)	
Transfer	8 (0.1%)	5 (0.1%)	3 (0.1%)	0 (0.0%)	0 (0.0%)	0 (0.0%)	0 (0.0%)	0 (0.0%)	0 (0.0%)	0 (0.0%)	
Voluntary discharge	2 (< 1%)	0 (0.0%)	2 (0.1%)	0 (0.0%)	0 (0.0%)	0 (0.0%)	0 (0.0%)	0 (0.0%)	0 (0.0%)	0 (0.0%)	
**Outcome of treatment**											
Deterioration	1 (< 1%)	0 (0.0%)	1 (< 1%)	0 (0.0%)	0 (0.0%)	0 (0.0%)	0 (0.0%)	0 (0.0%)	0 (0.0%)	0 (0.0%)	
Improvement	1,904 (24.8%)	761 (19.8%)	839 (31.7%)	12 (14.8%)	150 (20.7%)	48 (39.3%)	2 (13.3%)	0 (0.0%)	88 (43.3%)	4 (18.2%)	
Recovery	5,775 (75.2%)	3,088 (80.2%)	1,809 (68.3%)	69 (85.2%)	576 (79.3%)	74 (60.7%)	13 (86.7%)	13 (100%)	115 (56.7%)	18 (81.8%)	
Without changes	2 (<1%)	2 (0.1%)	0 (0.0%)	0 (0.0%)	0 (0.0%)	0 (0.0%)	0 (0.0%)	0 (0.0%)	0 (0.0%)	0 (0.0%)	
**Total**	**7,682**	**3,851**	**2,649**	**81**	**726**	**122**	**15**	**13**	**203**	**22**	

*Pearson’s chi-squared. ICD-10 codes: N80.0, Endometriosis of uterus; N80.1, Endometriosis of ovary; N80.2, Endometriosis of fallopian tube; N80.3, Endometriosis of pelvic peritoneum; N80.4, Endometriosis of rectovaginal septum and vagina; N80.5, Endometriosis of intestine; N80.6, Endometriosis in cutaneous scar; N80.8, Other endometriosis; N80.9, Endometriosis, unspecified. Outcome of stay terminology description: Discharge – patient went home after treatment; Transfer - patient was transferred to another hospital; Voluntary discharge – patient left a hospital before treatment completed due to personal demand; Death – patient death associated with treatment/surgery. Outcome of treatment terminology description: Without changes – patent was discharged without improvement; Recovery – patient was discharged with recovery; Improvement - patent was discharged with improvement; Deterioration - patent was discharged/transferred to another hospital with deterioration.

### 3.4 Endometriosis association with infertility

**FIGURE 5:**
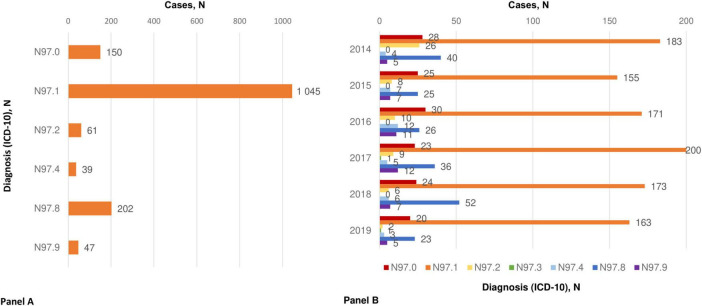
Endometriosis association with infertility. **(A)** Types of infertility associated with endometriosis; **(B)** distribution of endometriosis associated with infertility by years. ICD 10 codes: N97.0 - Female infertility associated with anovulation; N97.1 - Female infertility of tubal origin; N97.2 - Female infertility of uterine origin; N97.3 - Female infertility of cervical origin; N97.8 - Female infertility of other origin; N97.9 - Female infertility, unspecified.

Figure 5 shows endometriosis cases associated with infertility among the studied population. The most common type of infertility associated with cases of endometriosis was female infertility of tubal origin (ICD-10 code “N97.1”) – 67.7% of all infertility cases associated with endometriosis (Figure 5A). Other reported but less common cases were female infertility of another origin (ICD-10 code “N97.8”) and female infertility associated with anovulation (ICD-10 code “N97.0”).

The yearly distribution of endometriosis associated with infertility registered in the UNEHS is illustrated in Figure 5B.

### 3.5 Complications

**FIGURE 6:**
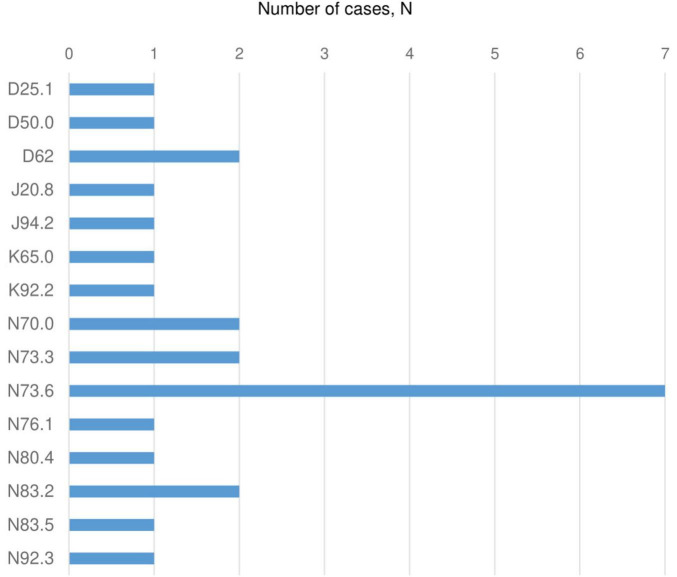
Complications of endometriosis (2014–2019). ICD 10 codes: D25.1 - Intramural leiomyoma of uterus; D50.0 - Iron deficiency anemia secondary to blood loss (chronic); D62 - Acute posthaemorrhagic anemia; J20.8 -Acute bronchitis due to other specified organisms; J94.2 – Hemotorax; K65.0 - Acute peritonitis; K92.2 - Gastrointestinal hemorrhage, unspecified; N70.0 -Acute salpingitis and oophoritis; N73.3 - Female acute pelvic peritonitis; N73.6 - Female pelvic peritoneal adhesions; N76.1 - Subacute and chronic vaginitis; N80.4 - Endometriosis of rectovaginal septum and vagina; N83.2 - Other and unspecified ovarian cysts; N83.5 - Torsion of ovary, ovarian pedicle and fallopian tube; N92.3 - Ovulation bleeding.

Complications in patients with endometriosis registered in the UNEHS are presented in Figure 6. The most common condition complicating endometriosis was female pelvic peritoneal adhesions (ICD-10 code “N73.6”). The other reported complications were unspecified ovarian cysts (ICD-10 “N83.2”) and acute posthaemorrhagic anemia (ICD-10 “D62”). Such complications as acute salpingitis and oophoritis (ICD-10 code “N70.0”), acute pelvic peritonitis (ICD-10 “N73.3”), acute posthaemorrhagic anemia (ICD-10 “D62”), and unspecified ovarian cysts (ICD-10 “N83.2) registered after surgical treatment were seen with the same rate. However, the overall reported number of complications was very low.

## 4 Discussion

Estimation of the endometriosis epidemiology is essential, as this gynecological condition is a major cause of infertility, chronic pelvic pain, and dysmenorrhea in many women (6, 13, 25). It is a significant health issue for reproductive-age women due to the necessity of specific medical and surgical management and associated fertility problems (6, 25). The Kazakhstani government prioritizes support of reproductive healthcare aiming to improve the birth rate and decrease maternal mortality (16, 17, 19, 26). For that, the State Program for the Development of Healthcare 2020–2025 has been approved with a budget of USD 7.5 billion (26). However, proper budget distribution and execution require a clear understanding of diseases’ prevalence and related health burdens. To date, no reliable statistics are available for endometriosis epidemiology in Kazakhstan. Therefore, this study’s aim was to investigate the epidemiology, complications, surgical management approaches, and outcomes of endometriosis in Kazakhstan.

### 4.1 Main findings and comparison with existing literature

In this study, the age of patients registered in the national electronic database with endometriosis ranged between 17 and 54 years, with an average age of 37 years. These data are comparable with the most recent studies on the epidemiology of endometriosis from Turkiye (27) and the United States of America (USA) (28) where the average age of women with endometriosis was 30 years old (range 18–50) and 37 years old (range 18–45), respectively. In the current study, the majority of women diagnosed with endometriosis were of reproductive age, between 25 and 44 years with the highest incidence in the 20–24, 25–29, and 30–34 years old age groups. These findings are in line with the results of the Australian study by Rowlands et al. (29), however, are in contradiction to the findings of the study from the USA by Christ et al. the incidence was highest among women aged 36–45 years (28).

This study revealed a low rate of endometriosis among adolescent patients and young women of in the 20–24 years old age group — 0.02 and 0.2 per 1,000 women of the corresponding age, that reflects to 0.8 and 6.8% incidence, respectively. This finding of our study is comparable and in agreement with the Australian study on the epidemiology of endometriosis where the rate of the disease was 0.2 per 1,000 persons of the same age (29) and with the findings of the Spanish study (30). However, low rates of endometriosis in adolescent patients and young women found in our study contradicts findings of the recent research by Zannoni et al. where the prevalence of endometriosis and adenomyosis in young women was 25.0 and 46.0%, respectively (31). Moreover, the prevalence of endometriosis in young women with dysmenorrhea is even higher (ranged between 25 and 73%) (32). On the other hand, similar to our findings, Zannoni et al. reported higher incidence of endometriosis among young women (20–24 years old group) than among adolescents (14–19 years old group). Thus, since endometriosis represents the main cause of secondary dysmenorrhea among adolescent and young women, the condition should be carefully managed as dysmenorrhea has a great impact on adolescents’ lives and future reproductive function (32).

In the study by Rowlands et al. the authors found a sharp increase (30-fold) in endometriosis incidence at age 30–34 years (6 compared to 0.2 per 1,000 women of the corresponding age), while in our study these findings are in contradiction to the compared study as the disease increased only 2-fold (0.4 compared to 0.2 per 1,000 women of corresponding age) among women of 35–39, 40–44, and 45–49 years old age groups. Rates of endometriosis reported by the study from the USA (29) are also higher than the results of our study (17.4–30.2 per 10,000 women in the USA vs. 0.2–0.4 per 1,000 women in Kazakhstan) (29). This can be explained by the underestimation of the endometriosis cases in Kazakhstan and the existing inaccuracy of the data registration in the Kazakhstani healthcare electronic system due to the recently introduced electronic healthcare system. The efforts on precise registration of healthcare data have to be reinforced in Kazakhstan.

Unfortunately, no studies on the epidemiology of endometriosis are available from Central Asian countries and/or post-Soviet countries with similar population and healthcare systems to compare the epidemiological indicators of the disease.

In this study, unequal incidence of endometriosis was found in the different regions of the country, which has no underling objective background. Moreover, regions with a larger population were found to have lower incidence of endometriosis. The distribution of endometriosis cases analyzed in the nationwide database reveals a huge difference in the number of cases registered in the different regions. This may be a result of improper registration. Thus, the accuracy of disease registration and reporting has to be improved to ensure proper management.

The number of patients registered with endometriosis from urban areas was much higher than that of the rural regions. This finding is in apparent agreement with the compared Spanish study on epidemiology of endometriosis, where incidence rates of the disease in women from rural areas were lower (30). There are two possible explanations for this fact. On one side, residents of rural areas are less exposed to the environmental toxins and pollutants playing role in the pathogenesis of endometriosis (33), thus have less risk factors. On the other side as studies report, population of rural and remote areas may have unequal access to medical care including gynecology specialists (16, 27).

Although the previous research on endometriosis outlined the estimated prevalence as around 10% (3, 8), a low prevalence of endometriosis was reported in the Kazakhstani UNEHS (0.12%). Despite the fact that this finding contradicts the overall trend on endometriosis prevalence, it is comparable with previous studies among the Spanish female population (30) where the prevalence of the disease was reported at the level of 0.7% or the USA population (28) with the reported low prevalence of endometriosis at the level of 1.9%. However, if compared with the recent study among women in Turkiye (27), our study population had significantly lower endometriosis prevalence (0.12%) than Turkish women where the disease is reported among 18.3% of the study subjects. These reported variations in prevalence between studies with low prevalence from the USA, Spain, and with high prevalence could be explained by differences in the studies’ design: the results of our study, study from the USA (28) and study from Spain (30) are based on the national electronic information systems reports, while the study from Turkiye (27) was based on self-reported surveys. The low prevalence of endometriosis reported in the Kazakhstani UNEHS clearly shows that the ICD-10 code utilization and overall disease-reporting arrangement should be improved in the frame of the local healthcare system. This finding is in line with assumption stating that endometriosis reporting and registration appears to be inaccurate, which leads to underestimation of the disease (6, 8).

In the current study endometriosis of the uterus, ovarian endometriosis, and endometriosis of pelvic peritoneum were the most prevalent types of endometriosis. These findings are in agreement with the investigation from the USA population-based study reporting data for the period of 2006–2015 (28, 29).

While researching the management of endometriosis, in this study laparoscopic cystectomy was found to be the most common procedure associated with ovarian endometriosis. This approach with laparoscopic management is in line with the most recent European Society of Human Reproduction and Embryology (ESHRE) guideline on endometriosis management (34). The other common procedures were hysteroscopy and hysteroscopy with biopsy performed for women with uterine endometriosis. This frequency of these surgical procedures is logical considering that endometriosis of the uterus and ovarian endometriosis were the most prevalent types of the disease. This is in line with the available sources, where researchers recommend operative hysteroscopy as a suitable option for cases of superficial adenomyosis as a treatment modality (35–37).

Data from UNEHS shows that female infertility is strongly associated with endometriosis of any localization, which is in agreement with previous reports (6, 13, 26–30).

Interestingly, in this study inflammatory complications of endometriosis appeared with the same rate as acute posthaemorrhagic anemia, which highlights the importance of proper infection prevention and application of hemostatic techniques during surgery. Furthermore, a recent research reported a strong interconnection between presence of endometriosis and recurrence of pelvic inflammatory diseases (38). Thus, early detection of inflammatory complications is essential to administer adequate treatment and facilitate the healing processes (39).

Generally, a very small number of complications are reported in the UNEHS among women with endometriosis and in association with the disease treatment. This could be related to the “punishment culture” that was present in the Kazakhstani national healthcare system, when any complication that happened with a patient was considered as a physician’s mistake even if it is a statistically prevalent and expected type of complication. This led to the development of specific “complication-hiding” culture when healthcare professionals did not report complications concerning negative impacts on their careers.

### 4.2 Study strengths and limitations

The main strength is novelty of this study, as this is the first one providing epidemiological data on the incidence, prevalence, complications, surgical management approach, and outcomes of endometriosis in Kazakhstan. In this investigation a large cohort of patients’ data are analyzed and covered the female population of Kazakhstan for the period of 6 years (2014–2019). Since the health-related records in the UNEHS were associated with the available socio-demographic information, this enabled to reduce missing data. This study also has some limitations mostly related to the electronic healthcare system design. UNEHS was established and introduced into the clinical practice in 2014, and is still under continuous development due to existing drawback requiring improvements (17, 23). Namely, the electronic system in its current form is not ideal as it does not have information on important socio-demographic data like education and income. Moreover, it does not save a woman’s marital status, gynecological anamnesis, parity history, symptoms and staging of endometriosis. It should be noted as another limitation that, compared to surgical management options, which are documented in the UNEHS and could be retrieved via ICD-9 codes, information on medical management of patients with endometriosis are not available in the national healthcare information system. Moreover, improvements in accuracy of coding from the healthcare professionals should be improved to adequately report different types of endometriosis and cases of deep endometriosis. For the further analysis of the data from 2023 and onward, the UNEHS is expected to be improved to provide these missing variables, which could facilitate the healthcare data analysis’ results and conclusions.

## 5 Conclusion

Endometriosis is a chronic, underestimated disease that according to the UNEHS affects 0.12% of Kazakhstani reproductive-age women. A huge proportion of women with endometriosis in Kazakhstan suffer from infertility. Analysis of the UNEHS reveals that there is an inconsistent and incomplete reporting and registration of endometriosis and its treatment, which affect the overall statistics on epidemiology and outcomes of the disease. Therefore, the data from the national healthcare electronic system does not reflect the endometriosis real burden. Gynecology specialists should be aware that the proper diagnosis of the disease would ensure provision of an adequate management. Establishing incidence and prevalence of endometriosis is an important initial step toward building a strong background for future research, which would improve knowledge on the disease etiology, pathogenesis, and progression, thus contribute to better management. Governmental health authorities and gynecology clinical specialists must work together to ensure the endometriosis proper diagnosis and registration. New treatment options recently approved for endometriosis should be applied.

## Data Availability

The original contributions presented in this study are included in this article/Supplementary material, further inquiries can be directed to the corresponding author.

## Footnotes

1
https://www.icd10data.com/

## References

[1] Terzic M Aimagambetova G Garzon S Bapayeva G Ukybassova T Terzic S Ovulation induction in infertile women with endometriotic ovarian cysts: current evidence and potential pitfalls. *Minerva Med*. (2020) 111:50–61. 10.23736/S0026-4806.19.06346-8 31755673

[2] Aimagambetova G Terzic S Bapayeva G Micic J Kongrtay K Laganà AS Endometriotic cyst and infertility. In: Duncan LT editor. *Advances in Health and Disease.* Vol. 38 New York, NY: Nova Science Publisher (2021). p. 31–67.

[3] Taylor HS Kotlyar AM Flores VA. Endometriosis is a chronic systemic disease: clinical challenges and novel innovations. *Lancet*. (2021) 397:839–52. 10.1016/S0140-6736(21)00389-5 33640070

[4] Terzic M Aimagambetova G Kunz J Bapayeva G Aitbayeva B Terzic S Molecular basis of endometriosis and endometrial cancer: current knowledge and future perspectives. *Int J Mol Sci*. (2021) 22:9274. 10.3390/ijms22179274 34502183 PMC8431548

[5] Bulun SE Yildiz S Adli M Chakravarti D Parker JB Milad M Endometriosis and adenomyosis: shared pathophysiology. *Fertil Steril.* (2023) 119:746–50. 10.1016/j.fertnstert.2023.03.006 36925057

[6] Koninckx PR Ussia A Adamyan L Tahlak M Keckstein J Wattiez A The epidemiology of endometriosis is poorly known as the pathophysiology and diagnosis are unclear. Best practice & research. *Clin Obstetr Gynaecol.* (2021) 71:14–26. 10.1016/j.bpobgyn.2020.08.005 32978068

[7] Bourdon M Santulli P Marcellin L Maignien C Maitrot-Mantelet L Bordonne C Adenomyosis: an update regarding its diagnosis and clinical features. *J Gynecol Obstetr Hum Reprod.* (2021) 50:102228. 10.1016/j.jogoh.2021.102228 34520877

[8] Sarria-Santamera A Orazumbekova B Terzic M Issanov A Chaowen C Asúnsolo-Del-Barco A. Systematic review and meta-analysis of incidence and prevalence of endometriosis. *Healthcare (Basel, Switzerland).* (2020) 9:29. 10.3390/healthcare9010029 33396813 PMC7824417

[9] Parazzini F Esposito G Tozzi L Noli S Bianchi S. Epidemiology of endometriosis and its comorbidities. *Eur J Obstet Gynecol Reprod Biol.* (2017) 209:3–7. 27216973 10.1016/j.ejogrb.2016.04.021

[10] Nowak-Psiorz I Ciećwież SM Brodowska A Starczewski A. Treatment of ovarian endometrial cysts in the context of recurrence and fertility. *Adv Clin Exp Med.* (2019) 28:407–13. 30659784 10.17219/acem/90767

[11] Somigliana E Vigano P Benaglia L Busnelli A Berlanda N Vercellini P. Management of endometriosis in the infertile patient. *Semin Reprod Med.* (2017) 35:31–7. 27926972 10.1055/s-0036-1597125

[12] Coccia ME Nardone L Rizzello F. Endometriosis and infertility: a long-life approach to preserve reproductive integrity. *Int J Environ Res Public Health.* (2022) 19:6162. 10.3390/ijerph19106162 PMC9141878 35627698

[13] Vercellini P Viganò P Bandini V Buggio L Berlanda N Somigliana E. Association of endometriosis and adenomyosis with pregnancy and infertility. *Fertil Steril.* (2023) 119:727–40. 10.1016/j.fertnstert.2023.03.018 36948440

[14] Vidali A Riccio LGC Abrao MS. Endometriosis and recurrent pregnancy loss: two manifestations of the same underlying dysfunction? *Fertil Steril.* (2023) 119:836–7. 10.1016/j.fertnstert.2023.03.012 36933642

[15] World Population Review Kazakhstan. (2023). Available at https://worldpopulationreview.com/countries/kazakhstan-population (accessed July 24, 2023).

[16] Sakko Y Aimagambetova G Terzic M Ukybassova T Bapayeva G Gusmanov A The prevalence, indications, outcomes of the most common major gynecological surgeries in Kazakhstan and recommendations for potential improvements into public health and clinical practice: analysis of the national electronic healthcare system (2014-2019). *Int J Environ Res Public Health.* (2022) 19:14679. 10.3390/ijerph192214679 36429398 PMC9690357

[17] Sakko Y Turesheva A Gaipov A Aimagambetova G Ukybassova T Marat A Epidemiology of spontaneous pregnancy loss in Kazakhstan: a national population-based cohort analysis during 2014–2019 using the national electronic healthcare system. *AOGS.* (2023) 102:1682–93. 10.1111/aogs.14669 PMC10619606 37667510

[18] National Agency for Statistics and Strategic Planning of the Republic of Kazakhstan. *Population count.* (2023). Available online at: https://stat.gov.kz/ru/ (accessed July 24, 2023).

[19] Issanov A Aimagambetova G Terzic S Bapayeva G Ukybassova T BAikoshkarova S Impact of governmental support to the IVF clinical pregnancy rates: differences between public and private clinical settings in Kazakhstan-a prospective cohort study. *BMJ Open.* (2022) 12:e049388. 10.1136/bmjopen-2021-049388 35165106 PMC8845187

[20] Bapayeva G Aimagambetova G Issanov A Terzic S Ukybassova T Aldiyarova A The effect of stress, anxiety and depression on in vitro fertilization outcome in Kazakhstani public clinical setting: a cross-sectional study. *J Clin Med.* (2021) 10:937. 10.3390/jcm10050937 33804325 PMC7975982

[21] Lokshin VN Khoroshilova IG Kuandykov EU. Personified approach to genetic screening of infertility couples in ART programs. *Report of the National Academy of Sciences of the Republic of Kazakhstan.* Vol. 1 (2018). p. 37–41.

[22] Aimagambetova G Issanov A Terzic S Bapayeva G Ukybassova T Baikoshkarova S The effect of psychological distress on IVF outcomes: reality or speculations? *PLoS One.* (2020) 15:e0242024. 10.1371/journal.pone.0242024 33315878 PMC7735622

[23] Gusmanov A Zhakhina G Yerdessov S Sakko Y Mussina K Alimbayev A Review of the research databases on population-based registries of unified electronic healthcare system of Kazakhstan (UNEHS): possibilities and limitations for epidemiological research and real-world evidence [published online ahead of print, 2022 Dec 7]. *Int J Med Inform.* (2022) 170:104950. 10.1016/j.ijmedinf.2022.104950 36508752

[24] StataCorp LLC. *Stata Statistical Software: Release 16.* College Station, TX: StataCorp LLC (2019).

[25] Guo SW. Various types of adenomyosis and endometriosis: in search of optimal management. *Fertil Steril.* (2023) 119:711–26. 10.1016/j.fertnstert.2023.03.021 36963717

[26] International Trade Administration Kazakhstan - Country Commercial Guide. (2023). Available online at: https://www.trade.gov/country-commercial-guides/kazakhstan-healthcare (accessed August 02, 2023).

[27] Yuksel Ozgor B Azamat S Berkay EG Türeli D Ozdemir I Topaloðlu S Epidemiology of endometriosis awareness in Turkey. *Cureus.* (2023) 15:e37536. 10.7759/cureus.37536 37193420 PMC10182833

[28] Christ JP Yu O Schulze-Rath R Grafton J Hansen K Reed SD. Incidence, prevalence, and trends in endometriosis diagnosis: a United States population-based study from 2006 to 2015. *Am J Obstetr Gynecol.* (2021) 225:.e1–500. 10.1016/j.ajog.2021.06.067 34147493

[29] Rowlands I Abbott J Montgomery G Hockey R Rogers P Mishra G. Prevalence and incidence of endometriosis in Australian women: a data linkage cohort study. *BJOG.* (2020) 128:657–65. 10.1111/1471-0528.16447 32757329

[30] Medina-Perucha L Pistillo A Raventós B Jacques-Aviñó C Munrós-Feliu J Martínez-Bueno C Endometriosis prevalence and incidence trends in a large population-based study in Catalonia (Spain) from 2009 to 2018. *Womens Health (Lond).* (2022) 18:17455057221130566. 10.1177/17455057221130566 36281527 PMC9608029

[31] Zannoni L Del Forno S Raimondo D Arena A Giaquinto I Paradisi R Adenomyosis and endometriosis in adolescents and young women with pelvic pain: prevalence and risk factors. *Minerva Pediatr.* (2024) 76:57–63. 10.23736/S2724-5276.20.05842-9 32549030

[32] Martire FG Piccione E Exacoustos C Zupi E. Endometriosis and adolescence: the impact of dysmenorrhea. *J Clin Med.* (2023) 12:5624. 10.3390/jcm12175624 37685691 PMC10488856

[33] Terzic S Kongrtay K Aimagambetova G Bapayeva G Micic J Laganà AS Environmental and occupational pollutants exposure and infertility. In: Duncan LT editor. *Advances in Health and Disease.* Vol. 38 New York, NY: Nova Science Publisher (2021). p. 123–46.

[34] Becker CM Bokor A Heikinheimo O Horne A Jansen F Kiesel L ESHRE endometriosis guideline group. ESHRE guideline: endometriosis. *Hum Reprod Open.* (2022) 2022:hoac009. 10.1093/hropen/hoac009 35350465 PMC8951218

[35] Sardo ADS Calagna G Santangelo F Zizolfi B Tanos V Perino A The role of hysteroscopy in the diagnosis and treatment of adenomyosis. *BioMed Res. Int.* (2017) 2017:1–7. 10.1155/2017/2518396 PMC5568620 28852646

[36] Gordts S Grimbizis G Campo R. Symptoms and classification of uterine adenomyosis, including the place of hysteroscopy in diagnosis. *Fertil Steril.* (2018) 109:380–8.e1. 10.1016/j.fertnstert.2018.01.006 29566850

[37] Gkrozou F Vatopoulou A Skentou C Paschopoulos M. Diagnosis and treatment of adenomyosis with office hysteroscopy-a narrative review of literature. *Diagnostics (Basel, Switzerland).* (2023) 13:2182. 10.3390/diagnostics13132182 37443576 PMC10340732

[38] Zografou Themeli M Nirgianakis K Neumann S Imboden S Mueller MD. Endometriosis is a risk factor for recurrent pelvic inflammatory disease after tubo-ovarian abscess surgery. *Arch Gynecol Obstetr.* (2023) 307:139–48. 10.1007/s00404-022-06743-6 36036826 PMC9422932

[39] Montanari E Reh LM Dauser B Birsan T Hudelist G. Serial assessment of inflammatory parameters for prediction of septic complications following surgery for colorectal endometriosis: a descriptive, retrospective study. *Wiener Klin Wochenschrift.* (2022) 134:118–24. 10.1007/s00508-021-01916-w 34338850 PMC8857128

