# Supplementary material for: Epidemiology of endometriosis in Kazakhstan: a national population-based cohort analysis (2014–2019) using data from the national electronic healthcare system
Source: Front Med (Lausanne). 2025 Jan 7;11:1436458. doi: 10.3389/fmed.2024.1436458 (PMC11746094; doi:10.3389/fmed.2024.1436458)
Supplement: Supplementary file 1 [file Table_1.docx]

| **Supplementary Table 1. Diagnoses of the study subjects by regions (2014-2019)** | | | | | | | | | | | |
| --- | --- | --- | --- | --- | --- | --- | --- | --- | --- | --- | --- |
| **Variable** | **Overall** | **Diagnosis (ICD-10), N (%)** | | | | | | | | | **p-value** |
|  |  | **N80.0** | **N80.1** | **N80.2** | **N80.3** | **N80.4** | **N80.5** | **N80.6** | **N80.8** | **N80.9** |  |
| Location |  | | | | | | | | | | <0.001* |
| Akmola region | 83 (1.1%) | 40 (1.0%) | 32 (1.2%) | 2 (2.5%) | 5 (0.7%) | 3 (2.5%) | 0 (0.0%) | 0 (0.0%) | 0 (0.0%) | 1 (4.5%) |  |
| Aktobe region | 37 (0.5%) | 4 (0.1%) | 31 (1.2%) | 0 (0.0%) | 1 (0.1%) | 0 (0.0%) | 0 (0.0%) | 0 (0.0%) | 1 (0.5%) | 0 (0.0%) |  |
| Almaty city | 1,092 (14.2%) | 379 (9.8%) | 565 (21.3%) | 4 (4.9%) | 80 (11.0%) | 13 (10.7%) | 0 (0.0%) | 0 (0.0%) | 48 (23.6%) | 3 (13.6%) |  |
| Almaty region | 138 (1.8%) | 36 (0.9%) | 97 (3.7%) | 2 (2.5%) | 2 (0.3%) | 0 (0.0%) | 0 (0.0%) | 0 (0.0%) | 1 (0.5%) | 0 (0.0%) |  |
| Astana city (capital) | 1,361 (17.7%) | 441 (11.5%) | 615 (23.2%) | 18 (22.2%) | 179 (24.7%) | 54 (44.3%) | 1 (6.7%) | 0 (0.0%) | 42 (20.7%) | 11 (50.0%) |  |
| Atyrau region | 63 (0.8%) | 22 (0.6%) | 38 (1.4%) | 1 (1.2%) | 2 (0.3%) | 0 (0.0%) | 0 (0.0%) | 0 (0.0%) | 0 (0.0%) | 0 (0.0%) |  |
| East-Kazakhstan region | 1,000 (13.0%) | 589 (15.3%) | 226 (8.5%) | 14 (17.3%) | 65 (9.0%) | 15 (12.3%) | 11 (73.3%) | 10 (76.9%) | 66 (32.5%) | 4 (18.2%) |  |
| Karaganda region | 445 (5.8%) | 61 (1.6%) | 245 (9.2%) | 13 (16.0%) | 110 (15.2%) | 10 (8.2%) | 0 (0.0%) | 2 (15.4%) | 4 (2.0%) | 0 (0.0%) |  |
| Kostanay region | 154 (2.0%) | 57 (1.5%) | 87 (3.3%) | 4 (4.9%) | 5 (0.7%) | 0 (0.0%) | 0 (0.0%) | 0 (0.0%) | 1 (0.5%) | 0 (0.0%) |  |
| Kyzylorda region | 27 (0.4%) | 21 (0.5%) | 4 (0.2%) | 0 (0.0%) | 2 (0.3%) | 0 (0.0%) | 0 (0.0%) | 0 (0.0%) | 0 (0.0%) | 0 (0.0%) |  |
| Mangystau region | 154 (2.0%) | 65 (1.7%) | 45 (1.7%) | 3 (3.7%) | 12 (1.7%) | 11 (9.0%) | 0 (0.0%) | 0 (0.0%) | 17 (8.4%) | 1 (4.5%) |  |
| North-Kazakhstan region | 1,826 (23.8%) | 1,737 (45.1%) | 28 (1.1%) | 6 (7.4%) | 50 (6.9%) | 2 (1.6%) | 1 (6.7%) | 0 (0.0%) | 2 (1.0%) | 0 (0.0%) |  |
| Pavlodar region | 286 (3.7%) | 55 (1.4%) | 201 (7.6%) | 2 (2.5%) | 16 (2.2%) | 8 (6.6%) | 0 (0.0%) | 0 (0.0%) | 3 (1.5%) | 1 (4.5%) |  |
| Shymkent city | 19 (0.2%) | 8 (0.2%) | 10 (0.4%) | 0 (0.0%) | 0 (0.0%) | 0 (0.0%) | 0 (0.0%) | 0 (0.0%) | 1 (0.5%) | 0 (0.0%) |  |
| Turkestan region | 191 (2.5%) | 84 (2.2%) | 91 (3.4%) | 4 (4.9%) | 11 (1.5%) | 1 (0.8%) | 0 (0.0%) | 0 (0.0%) | 0 (0.0%) | 0 (0.0%) |  |
| West-Kazakhstan region | 223 (2.9%) | 23 (0.6%) | 81 (3.1%) | 3 (3.7%) | 100 (13.8%) | 5 (4.1%) | 1 (6.7%) | 1 (7.7%) | 8 (3.9%) | 1 (4.5%) |  |
| Zhambyl region | 583 (7.6%) | 229 (5.9%) | 253 (9.6%) | 5 (6.2%) | 86 (11.8%) | 0 (0.0%) | 1 (6.7%) | 0 (0.0%) | 9 (4.4%) | 0 (0.0%) |  |
| **Total** | **7,682** | **3,851** | **2,649** | **81** | **726** | **122** | **15** | **13** | **203** | **22** |  |
| Table footnotes  * Pearson's chi-squared  *ICD-10 codes:* N80.0 - Endometriosis of uterus; N80.1 - Endometriosis of ovary; N80.2 - Endometriosis of fallopian tube; N80.3 - Endometriosis of pelvic peritoneum; N80.4 - Endometriosis of rectovaginal septum and vagina; N80.5 - Endometriosis of intestine; N80.6 - Endometriosis in cutaneous scar; N80.8 - Other endometriosis; N80.9 - Endometriosis, unspecified | | | | | | | | | | | |
